# Preparation and Characterization of a Preformed Polyampholyte Particle Gel Composite for Conformance Control in Oil Recovery

**DOI:** 10.3390/polym15204095

**Published:** 2023-10-15

**Authors:** Iskander Gussenov, Alexey Shakhvorostov, Aigerim Ayazbayeva, Nargiz Gizatullina, Alexey Klivenko, Sarkyt Kudaibergenov

**Affiliations:** 1 Institute of Polymer Materials and Technology, Microregion “Atyrau 1”, 3/1, Almaty 050019, Kazakhstan; iskander.gussenov@gmail.com (I.G.); ayazbayeva.aigerim@gmail.com (A.A.); nar.gn@bk.ru (N.G.); alexeyklivenko@mail.ru (A.K.); 2 Department of Chemical and Biochemical Engineering, Satbayev University, Almaty 050013, Kazakhstan; 3 Department of Human Ecology, Shakarim University of Semey, Semey 071412, Kazakhstan

**Keywords:** preformed particle gels (PPGs), reinforced hydrogels, polyampholyte, acrylamide, APTAC, AMPS, bentonite, swelling degree, swelling kinetic, Young’s modulus, mechanical properties

## Abstract

Preformed particle gels (PPGs) based on acrylamide (AAm), (3-acrylamidopropyl) trimethylammonium chloride (APTAC), and 2-acrylamido-2-methyl-1-propanesulfonic acid sodium salt (AMPS) were synthesized via conventional free radical copolymerization. The resultant PPGs of various compositions were characterized using FTIR spectroscopy, TG and DT analysis, and mechanical testing. The swelling behavior of PPGs depending on ionic strength, temperature, degree of crosslinking, and pH was also studied. The obtained results show that the swelling mechanism of PPGs is mainly due to the diffusion of the solvent. The mechanical properties of PPGs were improved by creating a composite polymer network by adding the clay mineral (bentonite) to the reaction mixture of monomers, which also makes it possible to control the Young’s modulus and the swelling degree of the samples.

## 1. Introduction

One of the most commonly used and economically effective techniques for controlling water mobility in oil reservoirs is the use of gel-based treatments [1]. They can be divided into in situ gels and preformed gels. In situ gels have been used extensively for conformance control over the past three decades and mostly consist of partially hydrolyzed polyacrylamide physically crosslinked by Cr^3+^ or Al^3+^ ions. Preformed particle gel (PPG) is a polymer particle of cross-linked superabsorbents (hydrogels) that have the ability to swell in brine [2]. The synthetic PPG protocol has been considered previously in the literature [3,4,5,6,7,8]. PPG often represents composite polymeric materials reinforced with clay minerals and possesses unique properties that make it a promising material for the conformance control of oil wells. Various types of PPG are micro- and millimeter-sized particles. Gels with small particles can be used to reduce water production and profile control in matrix reservoirs where permeability is less than one Darcy [9,10]. Large particle gels with dimensions from micro- to millimeters are useful for conformity control in fractured formations or channels with permeability greater than a few Darcy [11].

The main types of PPG consist of water-soluble monomers and a crosslinker. The addition of such co-monomers as sodium acrylate and/or 2-acrylamido-2-methyl-1-propanesulfonic acid sodium salt also increases the swelling capacity of PPG. The added clay minerals regulate the strength of the PPG. Most PPGs behave like standard polyelectrolytes and shrink in the presence of various salts containing brine water due to the screening of macroions by low-molecular-weight electrolytes. In this connection, it is crucial to design salt-tolerant PPGs. In contrast to conventional polyelectrolytes, polyampholytes exhibit a swelling of the polymer network with the addition of salts [12]. The ability of low-charge-density amphoteric copolymers and terpolymers to swell and be effective viscosity enhancers in high-salinity and high-temperature reservoirs plays a crucial role in the enhanced oil recovery (EOR) processes [13]. Polyampholytes, due to their salt and temperature resistance, are widely applied in enhanced oil recovery (EOR) as viscosifying agents where thickeners are required in brine solution [14]. Reinforced polyampholyte hydrogels combine the advantages of polymer matrices and mechanical strength as well as controlled porosity to effectively retain water and other chemical agents. As a result, such materials can increase oil well productivity and reduce productivity losses. The present communication is devoted to the preparation and characterization of a series of crosslinked amphoteric terpolymers based on acrylamide (AAm), (3-acrylamidopropyl) trimethylammonium chloride (APTAC), and 2-acrylamido-2-methyl-1-propanesulfonic acid sodium salt (AMPS) for their potential application as conformance control agents.

## 2. Materials and Methods

### 2.1. Materials

For the synthesis of polyampholyte hydrogels, the following chemicals were used: anionic monomer—2-acrylamido-2-methyl-1-propanesulfonic acid sodium salt (AMPS, 50 wt.%); cationic monomer—3-acrylamidopropyltrimethylammonium chloride (APTAC, 75 wt.%); and uncharged monomer—acrylamide (AAm, 99% purity). N,N’-methylenebisacrylamide (MBAA, 99% purity) was used as a crosslinking agent. The polymerization initiator was ammonium persulfate (APS, 98% purity). The listed reagents were products of Sigma-Aldrich (USA) and were used without further purification. To improve the mechanical properties, a mineral filler—bentonite (BENTOLUX API, Almetyevsk, Tatarstan, Russia)—was used. Oilfield brines, natural solutions mostly containing sodium and calcium chloride, were supplied from the Uzen and Kalamkas oilfields located in western Kazakhstan. The total dissolved solids (TDS) of the Uzen and Kalamkas brines were equal to 61 and 129 g/L (Table 1).

### 2.2. Synthesis of Hydrogels Based on AAm-APTAC-AMPS

PPGs of various compositions, specifically [AAm]:[APTAC]:[AMPS] = 90:5:5, 80:10:10, 70:15:15, and 60:20:20 mol.%, herein abbreviated as AAm_90_-APTAC_5_-AMPS_5_, AAm_80_-APTAC_10_-AMPS_10_, AAm_70_-APTAC_15_-AMPS_15_, and AAm_60_-APTAC_20_-AMPS_20_, were synthesized via conventional free radical copolymerization (Table 2). The required amounts of monomers (AAm, APTAC, and AMPS), MBAA, and bentonite were dissolved in deionized water with constant stirring. Furthermore, the required amount of initiator, APS, was added to the solution and stirred until complete dissolution. The solution containing dissolved monomers, a crosslinking agent, mineral filler, and initiator was then transferred to a vial (diameter 1.5 cm, height 7 cm) and purged with Ar for 15–20 min to remove the dissolved oxygen. Then, the vial was carefully transferred into the thermostat. Free radical copolymerization was carried out at 60 °C for 3 h with constant stirring of the solution. To remove unreacted components and reach a fully swelled state, all synthesized composite gels were immersed in distilled water for a week during daily water changes.

### 2.3. Fourier-Transform Infrared Spectroscopy (ATR-FTIR) of Hydrogels

Functional groups of hydrogels were characterized with the help of Cary 660 FTIR spectroscopy (Agilent, Santa Clara, CA, USA) equipped with the Pike MIRacle ATR accessory (Attenuated Total Reflection mode). To measure the FTIR spectra, the hydrogels were swollen in 10 mL of deionized water and then freeze-dried for 24 h until moisture was removed. The FTIR spectra were measured at room temperature within the 700–4000 cm^−1^ range of wavenumbers.

### 2.4. Thermogravimetric Analysis (TGA) of Hydrogels

The thermogravimetric analysis of freeze-dried hydrogels was carried out using a LabSys Evo device (Setaram, Caluire-et-Cuire, France) in the temperature range 25–600 °C (heating rate: 10 °C·min^−1^) under an inert atmosphere. The maximum decomposition temperature of the hydrogels was determined from the differential thermal analysis (DTA) curve.

### 2.5. Analysis of Mechanical Properties of Hydrogels

Mechanical properties of the as-prepared hydrogels were studied using Texture/Mechanical Analyzer TA.XTplus Stable Micro Systems (Mason Technology, Surrey, UK) with the method described in [15]. Each hydrogel sample was measured 3 times at room temperature and the results were averaged. The stress–strain diagrams of hydrogels were obtained in compression mode. A cylindrical probe P/75 made of stainless steel with a diameter of 75 mm was used to press on the hydrogel sample and track the change in the compression force as a function of distance/time. The measurement parameters were cylindrical probe pre-test speed 1 mm/s, test speed 0.5 mm/s, trigger force 0.2 g, and remote target mode with a distance of 1.5 mm. The stress value (force per unit area) was calculated from the maximum force value:

(1) $$\mathrm{Stress}=\frac{\mathrm{Force}\;\left(g\right)}{\mathrm{Area}({\mathrm{mm}}^{2})}$$

Since the stress curve is proportional to the strain, the slope follows Young’s modulus. This means that in this region, the gel material underwent only elastic deformation [16].

### 2.6. Determination of Hydrogels’ Water-Holding Capacity at 25 °C

The swelling degrees of hydrogels were measured in deionized water, aqueous solutions of NaCl and CaCl_2_, acetate, and borate buffers. The swelling degree of hydrogels was determined using the conventional gravimetric method. A pre-weighed piece of dry hydrogel was immersed in a swelling medium at 25 °C and allowed to swell. After an adjusted period of time (3 days), the swollen hydrogels were quickly removed from the swelling medium (excess water was removed by blotting the sample with filter paper) and weighed. The swelling degree (SD) was calculated as follows:

(2) $$\mathrm{SD}=\frac{W_{s}-{\;W}_{d}}{W_{d}}$$

where W_s_ is the weight of the swollen hydrogel and W_d_ is the weight of the dry hydrogel.

### 2.7. Determination of Hydrogels’ Water-Holding Capacity in Aqueous Solution at Various Temperatures

The pre-weighed piece of dry hydrogel was immersed in water to swell at 30, 40, 50, 60, 70, and 80 °C (time interval was 24 h). After each interval, the swollen hydrogels were quickly removed from the swelling medium (excess water was removed by blotting the sample with filter paper) and weighed. The SD was calculated using Equation (2).

### 2.8. Determination of Water Holding Capacity and Water Absorption Kinetics of Hydrogels in Low-, Medium-, and High-Salinity Formation Water

Measurements of the SD and swelling kinetics of hydrogels were carried out in the formation waters of the Kalamkas and Uzen oil fields of Kazakhstan. The SD of hydrogels was also determined using the gravimetric method, as described above. A pre-weighed piece of dry hydrogel was immersed in the formation water to swell at 25 °C. At definite intervals, the swollen hydrogels were quickly removed from the swelling medium (excess water was removed by blotting the sample with filter paper) and weighed. The degree of SD was calculated using Equation (2).

### 2.9. Selectivity of Water Holding Capacity in the Water–Organic Mixture

As-prepared hydrogels were placed into distilled water for 3 days to remove unreacted components. Next, weighed samples of swollen hydrogels (about 10 mg) were immersed into water–acetone or water–ethanol mixtures with different amounts of organic solvents in test tubes, also for 3 days at 25 °C. After a specified period of time, the hydrogel samples were extracted from the water–organic mixtures, the residual moisture was removed with filter paper, and samples were weighed. The swelling degree of hydrogels in an aqueous organic mixture (${\mathrm{SD}}_{\mathrm{Org}}$) was determined using Equation (3):

(3) $${\mathrm{SD}}_{\mathrm{Org}}=\frac{m_{\mathrm{org}}}{m_{w}}$$

where m_org_ is the weight of the hydrogel in the water–organic solvent mixture and m_w_ is the weight of hydrogel swollen in water.

### 2.10. Scanning Electron Microscopy (SEM) of Hydrogels

For detailed morphological analyses, SEM images of the freeze-dried hydrogels were taken. Hydrogel samples were pre-washed in deionized water for 3 days. Next, the hydrated hydrogels were frozen and lyophilized using a Labconco FreeZone freeze-drying system (Geneva, Switzerland). The analysis of the internal structure was carried out using a MIRA 3 LMU scanning electron microscope (Tescan, Brno, Czech Republic) at an accelerating voltage of 20 kV.

## 3. Results and Discussions

**Figure 1:**
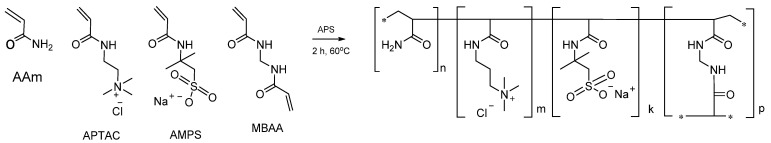
Schematic representation of the copolymerization of AAm, APTAC, and AMPS monomers.

Hydrogels of various compositions, with [AAm]:[APTAC]:[AMPS] ratios of 90-5-5; 80-10-10; 70-15-15; and 60-20-20 mol.% (abbreviated as Aam_90_-APTAC_5_-AMPS_5_, Aam_80_-APTAC_10_-AMPS_10,_ Aam_70_-APTAC_15_-AMPS_15_, Aam_60_-APTAC_20_-AMPS_20_), were synthesized via conventional free-radical-initiated copolymerization (Figure 1).

**Figure 2:**
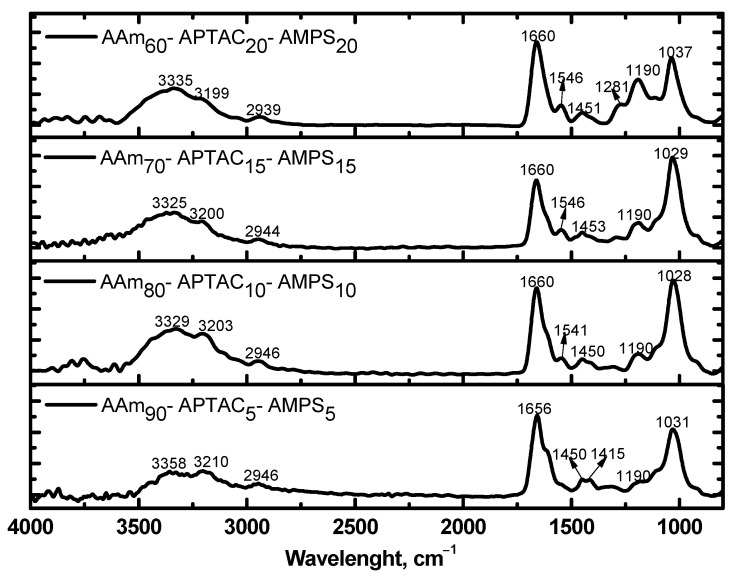
FTIR spectra of AAm-APTAC-AMPS hydrogels of various compositions.

Figure 2 shows the FTIR spectra of AAm-APTAC-AMPS hydrogels, which were similar due to the presence of the same monomers. The broad absorption band in the region of 3150–3400 cm^−1^ corresponded to the primary and secondary amide groups. The absorption bands in the region of 2800–3000 cm^−1^ were responsible for the asymmetric and symmetric vibrations of the CH groups. The absorption bands at 1550–1660 cm^−1^ belonged to the vibrations of the N-substituted groups, which were the amide I and amide II groups, respectively. The absorption band at 1450–1453 cm^−1^ was the characteristic medium peak of the C-H scissor vibration. The absorption band at 1415–1453 cm^−1^ corresponded to stretching vibrations of the C-N groups. The bending vibrations of the CH groups appeared at 1185–1190 cm^−1^. Finally, the absorption band in the region of 1020–1040 cm^−1^ corresponded to the S=O groups in AMPS units.

**Figure 3:**
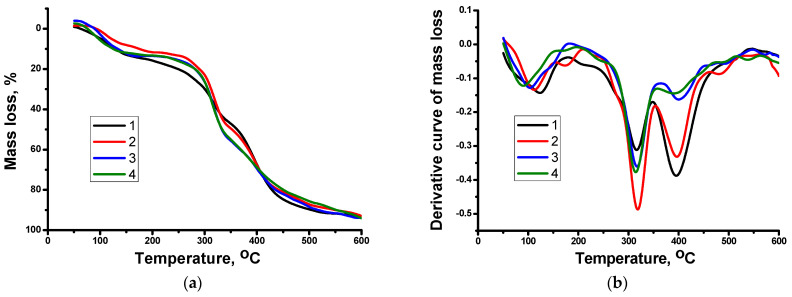
(**a**) TGA and (**b**) DTA curves of thermal decomposition of (1) Aam_90_-APTAC_5_-AMPS_5_, (2) Aam_80_-APTAC_10_-AMPS_10_, (3) Aam_70_-APTAC_15_-AMPS_15_, and (4) AAm_60_-APTAC_20_-AMPS_20_ hydrogels.

The results of thermogravimetric and differential thermal analyses of Aam-APTAC-AMPS hydrogels are shown in Figure 3a,b.

Dried hydrogels showed a 15–20% weight loss associated with the removal of residual water in the region of ~150 °C. The beginning of the thermal decomposition of hydrogels was observed at 260 °C. The complete thermal decomposition of AAm-APTAC-AMPS hydrogels occurred in the temperature range between 425 and 447 °C.

The mechanical properties of the as-prepared gels were studied to demonstrate the strength of the polyampholyte hydrogels reinforced with clay particles and to investigate the effect of composition on their properties (Table 3).

**Figure 4:**
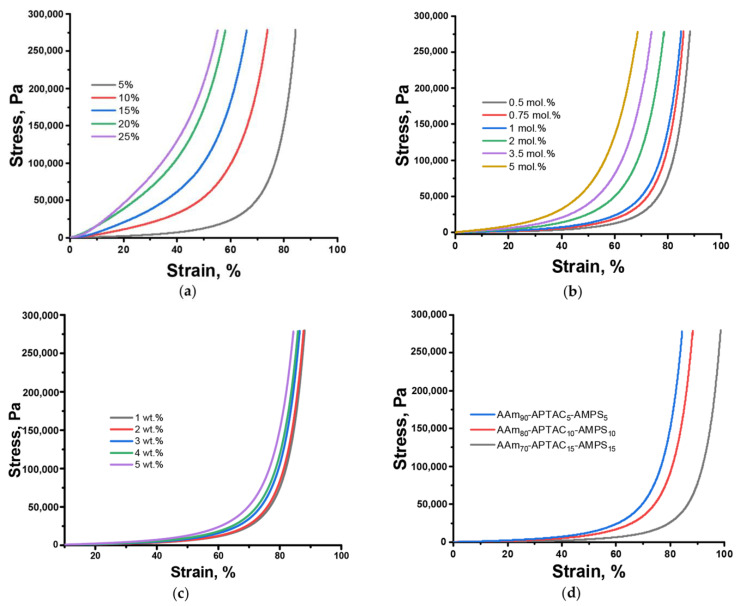
Stress–strain diagrams of AAm-APTAC-AMPS polyampholyte hydrogels at different (**a**) concentrations of monomers in the feed, (**b**) contents of cross-linker MBAA, (**c**) amounts of clay bentonite mineral, and (**d**) monomer composition.

In the case of different monomer concentrations, the elastic modulus (Young’s modulus) of AAm-APTAC-AMPS composite hydrogels increased from 173.7 to 2694.3 Pa with higher monomer content in the feed mixture (Figure 4a), which means that the samples became harder. The strength of the hydrogels was directly influenced by the crosslink density within their network (Figure 4b), which is directly related to the monomer concentration. At higher monomer concentrations, more reactive sites were available for crosslinking reactions during polymerization. Consequently, more covalent bonds were formed between the polymer chains, leading to a denser crosslinked network structure. This increased density of crosslinks resulted in stronger physical integrity, enabling the hydrogel to withstand mechanical stress and resist deformation. As a result, the Young’s modulus increased from 124 to 617 Pa. With an increase in monomer concentration and cross-linker in the feed, the final hydrogel had a smaller average mesh size, which referred to the spaces between the crosslinked polymer chains. A smaller mesh size corresponds to a tighter network, restricting the movement of water within the hydrogel matrix [17].

The presence of natural clay minerals, such as bentonite, during the synthesis of polyampholyte hydrogels significantly affects their mechanical properties. Bentonite is widely used in the oil and gas industry as a reinforcing filler in different formulations due to its unique properties, such as large surface area. Different amounts of bentonite present in the hydrogel matrix affected the mechanical properties of the resulting hydrogel (Figure 4c) [18]. First, the addition of bentonite increased the overall stiffness and Young’s modulus of the hydrogel from 82 to 173 Pa. This can be attributed to the strong interactions between the clay particles and the polymer chains, which act as physical crosslinkers and reinforce the three-dimensional network structure of the hydrogel. Second, bentonite particles acted as stress-relieving agents that effectively distributed and absorbed the mechanical forces acting on the polyampholyte hydrogel matrix. Third, as the bentonite concentration rose, the hydrogel’s swelling ratio could decrease, as clay particles reduced the polymer’s capacity for physical swelling and expansion and lowered the polymer content of the material as a function of mass.

When the concentration of both positively and negatively charged monomers increased, it led to an increase in the density of ionic crosslinks within the hydrogel network. The electrostatic interaction between positive and negative charges promoted the formation of strong physical crosslinks, resulting in a more robust and mechanically resistant hydrogel (Figure 4d) [19].

Overall, PPG made from reinforced polyampholyte hydrogel should not be very tough because swollen PPG particles are preferably elastic and deformable and move more easily through porous media under oilfield reservoir conditions [20].

Since hydrogels have the ability to retain water due to their three-dimensional (3D) polymer network, the swelling capacity and swelling rate of hydrogels depend on various parameters such as ionic strength, temperature, degree of crosslinking of the gel structure, type of monomeric groups, pH, etc. Various kinetic models for swelling rate have been presented, but most of them describe only diffusion or polymer relaxation as the predominant process for swelling rate.

Swelling kinetics are typically studied using the well-known Peppas model [21], but this model does not cover the entire interval of the swelling period and can only be used for a limited value of swelling degree. Recently, Yavari and Azizian [22] proposed a new model to describe the kinetics of hydrogel swelling. The new mixed model allowed us to describe the process of swelling in the whole range of values of the degree of swelling and evaluate whether it is a diffusion or relaxation process.

The model is based on a simple Equation (4) that takes into account the kinetics of swelling while allowing an evaluation of the effects of diffusion and relaxation of the polymer:

(4) $$S_{t}=S_{e}\left[1-e^{\left(-k_{1}t-k_{2}t^{1/2}\right)}\right]$$

where $S_{t}$ is the water-holding capacity of the hydrogel (g·g^−1^) at time T, $S_{e}$ is the equilibrium water-holding capacity of the hydrogel (g·g^−1^), and $k_{1}$ and $k_{2}$ are rate constants of the swelling.

**Figure 5:**
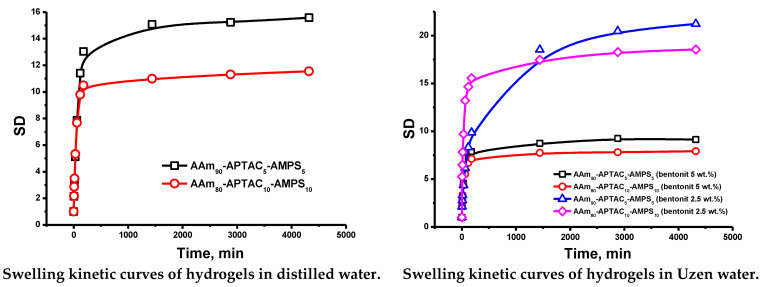

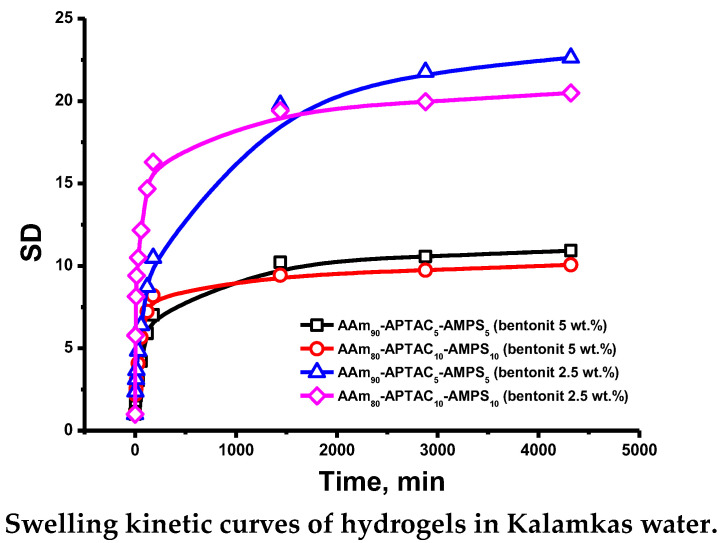
The swelling kinetic curves of reinforced polyampholyte hydrogels, plotted according to the model developed by Yavari and Azizian.

The swelling kinetic curves of the hydrogel are shown in Figure 5. It is clear that the model developed by Yavari and Azizian works well because the points in the curve lie with high precision (R^2^ = 0.99). If $k_{1}\;$>>$\;k_{2}$, then the swelling rate of the gels is determined by the relaxation of polymer chains; in the opposite case, $k_{1}\;$<<$\;k_{2}$, the swelling rate is determined by diffusion. If the values of *k*_1_ and *k*_2_ are close to each other, then the swelling rate is determined by both diffusion and relaxation modes. Table 4 summarizes the swelling kinetics of amphoteric hydrogels. The data indicate that the higher AAm content led to a slight increase in the SD of hydrogels. This phenomenon can be explained by the low content of oppositely charged units in the copolymers, which provide little crosslinking of the polymer chains [23]. The obtained data show that the mechanisms of the swelling process in pure and brine water are the same and mainly based on the diffusion process.

The presence of anionic and cationic groups in polyampholyte hydrogels should protect them from collapse in saline water. Normally, the SD of hydrogels decreases dramatically in solutions with high ionic strength due to the equalization of osmotic pressure between the gel and the solution [24]. Table 4 shows that the SD of hydrogels slightly decreased in Kalamkas and Uzen water. Here, the swelling mechanism was determined by the diffusion process and not by the relaxation of the chains. The table shows that in the case of hydrogel AAm80-APTAC10-AMPS10 (bentonite 5 wt.%) in pure water, the value of k2 was only 5 times higher than the value of k1. In this regard, we have concluded that the swelling rate of this sample was controlled by both processes, but by diffusion to a greater extent. Previously, it was shown that the presence of a low molecular weight electrolyte in solution reduces the electrostatic attraction between positively and negatively charged monomers by shielding anions and cations in the polymer chain, resulting in polymer chain expansion. This phenomenon is called the antipolyelectrolyte effect [25].

**Figure 6:**
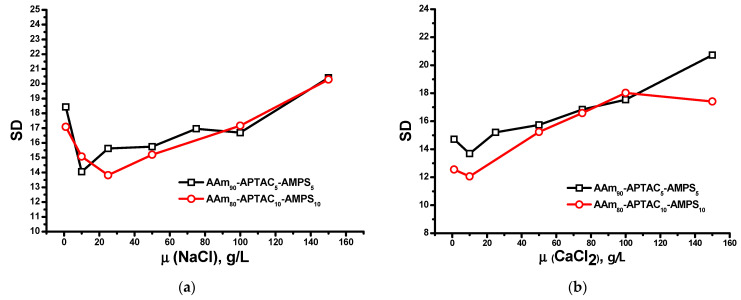
Effect of changing (**a**) NaCl and (**b**) CaCl_2_ concentrations on the swelling degree of hydrogels (bentonite concentration is 5 wt.%).

Figure 6 shows the effect of salt concentration on the SD of reinforced polyampholyte hydrogels. At low concentrations of NaCl, up to 10 g/L, the SD decreased. However, it increased in the range of NaCl concentration from 20 to 150 g/L. In the case of the CaCl_2_ solution, a constant increase in the SD was observed over the entire concentration range. The obtained results are in good agreement with the data described by Gao et al. [26].

The swelling behavior of the hydrogel was also affected by bentonite concentration. Increasing the bentonite content from 1 to 5 wt.% led to a more than two-fold decrease in SD. This is probably due to the fact that bentonite played the role of an additional crosslinker and hindered water absorption by the clay particles. Similar results were observed by Cheng et al. [24].

**Figure 7:**
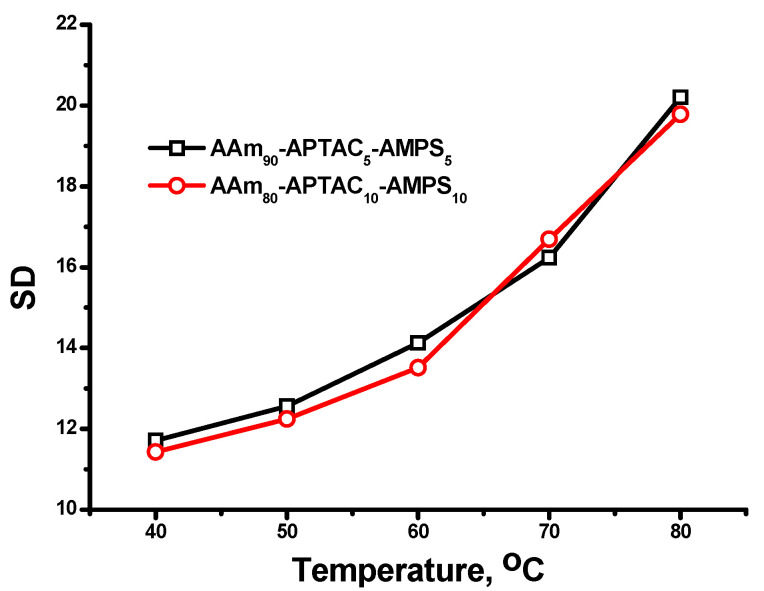
Effect of solution temperature on swelling degree of reinforced polyampholyte hydrogel.

The swelling responses of reinforced polyampholyte hydrogel AAm_90_-APTAC_5_-AMPS_5_ and AAm_80_-APTAC_10_-AMPS_10_ with respect to solution temperature are displayed in Figure 7. In general, for non-thermoresponsive hydrogels, an increase in temperature leads to an increase in the SD. This phenomenon is primarily due to the thermally induced kinetic energy of the water molecules, which accelerates their diffusion into the hydrogel matrix [27]. The increased diffusion rate of water molecules allows them to penetrate the polymer chains better, resulting in greater water absorption and swelling. A rise in the temperature weakens the interactions between polymer chains, promoting the relaxation and expansion of the polymer chains. This further facilitates water penetration into the hydrogel, increasing swelling capacity. Overall, the increased water absorption and expansion of the volume and structure of the hydrogel have significant effects on the potential application of such materials in the injection of preformed particle gels for conformance control in oil reservoirs.

**Figure 8:**
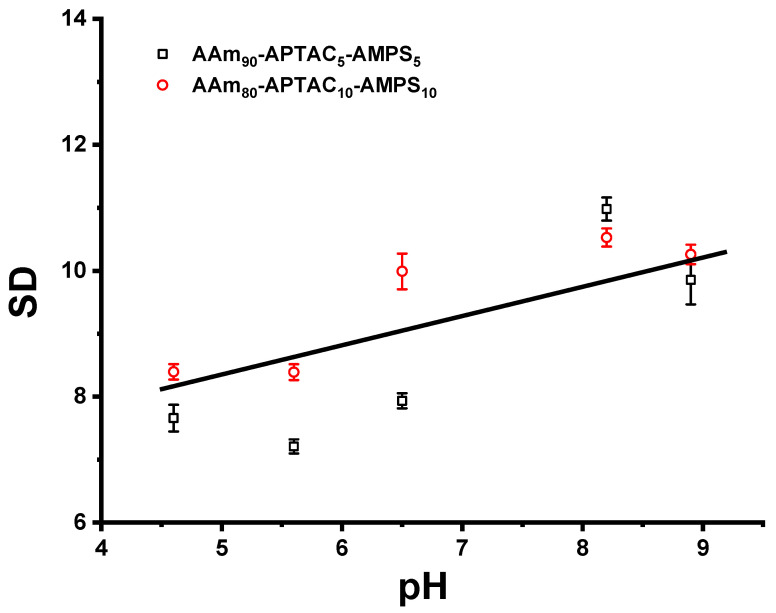
Influence of the pH values on the swelling degree of reinforced polyampholyte hydrogels.

pH values of brine water in Kazakhstan oil fields usually range between 6 and 9. The pH range may vary slightly depending on the mineral composition of the oil reservoir, the salt concentration, etc. It is commonly accepted that quenched polyampholytes, including AAm-APTAC-AMPS, contain strongly charged cationic and anionic monomers and retain their respective charges independently of pH [28]. As seen in Figure 8, the pH-dependent swelling degree of acrylamide-based low-charge polyampholytes AAm-APTAC-AMPS was in the range of 8.5 ± 1.5 g/g. The reason is that AAm-APTAC-AMPS polyampholytes contain fully ionized groups and changing the pH leads only to an ion exchanging process. The mutual compensation of positively and negatively charged groups of polyampholytes corresponds to a quasi-neutral state that is defined as the isoelectric point (IEP). In our case, the IEP could not be detected for AAm-APTAC-AMPS polyampholytes due to the fully ionized quaternary ammonium groups of APTAC (pH independent group) and the sulfo groups of AMPS in the polymer chain. Thermo-, pH-, and salt-responsive properties of amphoteric hydrogels developed in this work can be of help to predict the volume behavior of gels under oilfield conditions.

**Figure 9:**
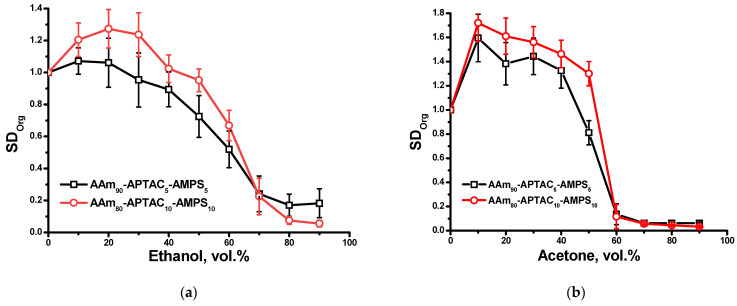
Swelling and deswelling behavior of AAm-APTAC-AMPS hydrogels in (**a**) water–ethanol and (**b**) water–acetone mixtures.

Earlier, the temperature [29], pH [30], and solvent-responsive [31] properties of polyampholyte gels were described. In this study, the influence of a water–ethanol and water–acetone mixture on the behavior of the AAm-APTAC-AMPS hydrogels was evaluated. Preliminary weighed hydrogels were placed into water–organic solvent mixtures for 3 days. Figure 9 demonstrates the dependence of the SD_Org_ on the volume fraction of ethanol or acetone in the mixture. The addition of up to 40 vol.% of ethanol or acetone led to significant swelling of the hydrogel samples. This is probably due to the improvement of the thermodynamic quality of the water–organic solvent mixture with respect to both hydrophobic and hydrophilic groups of polyampholytes, leading to better swelling of macromolecular chains. Further increasing the content of organic solvents to more than 50 or 60 vol.% leads to the gradual or sharp shrinking of hydrogel samples. This phenomenon can be explained by decreasing the dielectric permittivity of the water–organic solvent mixture with respect to ionic groups of macromolecules. In pure ethanol and acetone, the polyampholyte gel collapsed due to the significant suppression of the ionization state and the condensation of macroions and counterions due to poor thermodynamic quality, together with the low dielectric permittivity of solvents. Collapsing polyampholyte gels in organic solvents are important for removing blockages in the oil reservoir in the case of an emergency.

**Figure 10:**
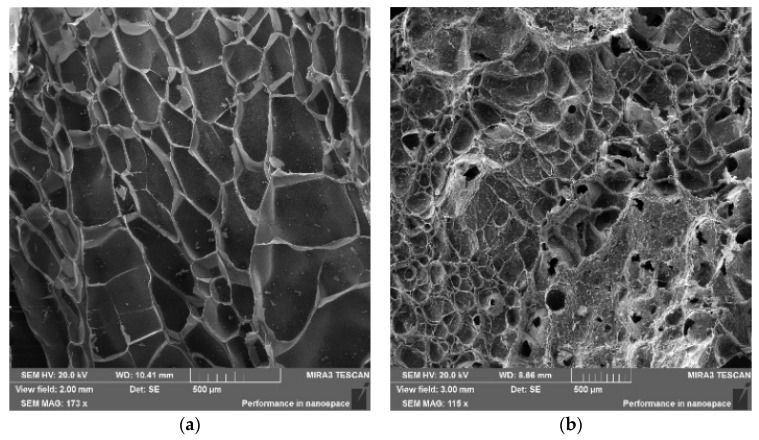
SEM images of AAm_90_-APTAC_5_-AMPS_5_ composite polyampholyte hydrogels crosslinked by (**a**,**b**) 1 mol.% MBAA in (**a**) the absence and (**b**) the presence of 5 wt.% bentonite.

The results of scanning electron microscopy (SEM) of the composite polyampholyte hydrogels crosslinked with 1 mol.% MBAA are shown in Figure 10a,b. The pore size of the pristine hydrogel without bentonite addition varied on average between 50 and 70 and 350 and 400 µm (Figure 10a). However, in the presence of bentonite, the average pore size of the polymer network decreased to 70–120 µm (Figure 10b). This is probably due to the fact that the bentonite played the role of an additional crosslinker and increased the mechanical properties of the composite hydrogels.

## 4. Conclusions

The effects of the synthesis parameters on the behavior of composite polyampholyte hydrogels AAm-APTAC-AMPS in deionized water, salty water, and water–organic solvent mixtures were investigated for the first time. The concentration of monomers affected the mechanical strength of hydrogels, and the gel strength could be improved up to 15 times if the monomer concentration was raised from 5 to 25%. The elastic modulus of hydrogels changed from 124 to 617 Pa depending on the crosslinker concentration in the range of 0.5 to 5 mol%. The mechanical strength was increased from 82.3 Pa to 173.7 Pa by adding mineral bentonite up to 5 wt%.

In the brine water, the swelling behavior of AAm- APTAC-AMPS -bentonite hydrogels demonstrated a good water uptake ability. The model of Yavari and Azizyan well describes the swelling kinetics of hydrogels. The migration of solvent molecules into the polymer network controlled the swelling mechanism. While the SD of hydrogels did not appreciably alter at a pH range between 4.5 and 8.0, the SD of polyampholyte gels heated to 80 °C doubled.

AAm-APTAC-AMPS hydrogels have good mechanical characteristics, are less sensitive to pH and brine water in oil reservoirs, and can expand in hot reservoirs and contract in organic solvents. All these properties are suitable for the use of these new materials for application in conformance control and oil recovery.

## Figures and Tables

**Table 1:** Brine composition.

Parameter/Element	Uzen Water	Kalamkas Water
pH	7.2	6.6
Density, g/cm^3^	1.043	
Ca^2+^, mg/L	3206.4	3200
Mg^2+^, mg/L	1094.4	2160
Na^+^ and K^+^, mg/L	18,816.3	43,789.4
Cl^-^, mg/L	37,605.4	79,566.2
SO_4_^2−^, mg/L	80.7	Not detected
CO_3_^2−^, mg/L	Not detected	Not detected
HCO^3−^, mg/L	341.6	158.6
Total salinity, mg/L	61,144.8	128,874.4
Hardness, mg-eq/L	250	-

**Table 2:** The composition of bentonite-reinforced polyampholyte hydrogels derived from Aam-APTAC-AMPS monomers.

№	AAm, mol.%	APTAC, mol.%	AMPS, mol.%	MBAA, mol.%	Bentonite, wt.%	APS, mol.%	Monomer Concentration, %
1	90	5	5	1	5	0.1	5
2							10
3							15
4							20
5							25
6	90	5	5	0.5	5	0.1	5
7				0.75			
8				1			
9				2			
10				3.5			
11				5			
12	90	5	5	1	1	0.1	5
13					2		
14					3		
15					4		
16					5		
17	90	5	5	1	5	0.1	5
18	80	10	10				
19	70	15	15				
20	60	20	20				

**Table 3:** Dependence of Young’s modulus on various parameters.

**Dependence of Young’s Modulus on Monomer Concentration (%)**	**Young’s Modulus, Pa**
5	173.7 ± 3.0
10	787.4 ± 42.0
15	1462.3 ± 76.0
20	2391.1 ± 93.0
25	2694.3 ± 306.0
**Dependence of Young’s modulus on MBAA concentration (mol.%)**	**Young’s Modulus, Pa**
0.5	124.0 ± 6.0
0.75	118.8 ± 8.0
1	173.7 ± 3.0
2	307.0 ± 10.0
3.5	356.6 ± 7.0
5	617.0 ± 9.0
**Dependence of Young’s modulus on bentonit concentration (wt.%)**	**Young’s Modulus, Pa**
1	82.3 ± 1.0
2	87.8 ± 5.0
3	104.7 ± 2.0
4	119.4 ± 8.0
5	173.7 ± 3.0
**Dependence of Young’s modulus on molar ratio of AAm-APTAC-AMPS**	**Young’s Modulus, Pa**
AAm_90_-APTAC_5_-AMPS_5_	173.7 ± 3.0
AAm_80_-APTAC_10_-AMPS_10_	147.3 ± 12.0
AAm_70_-APTAC_15_-AMPS_15_	88.9 ± 8.0
AAm_60_-APTAC_20_-AMPS_20_	51.4 ± 15.0

**Table 4:** Swelling kinetics parameters of reinforced polyampholyte hydrogels.

Sample	$S_{e}$	$k_{1}$	$k_{2}$	R^2^	Main Process
AAm_90_-APTAC_5_-AMPS_5_ in water (bentonite 5 wt.%)	15.32	7.60 × 10^−4^	0.0373	0.99	Diffusion
AAm_80_-APTAC_10_-AMPS_10_ in pure water (bentonite 5 wt.%)	11.28	0.0110	0.0589	0.99	Diffusion and relaxation
AAm_90_-APTAC_5_-AMPS_5_ in brine water from Uzen (bentonite 2.5 wt.%)	21.70	2.33 × 10^−4^	0.0416	0.99	Diffusion
AAm_90_-APTAC_5_-AMPS_5_ in brine water from Uzen (bentonite 5 wt.%)	9.03	0.0035	0.1052	0.98	Diffusion
AAm_80_-APTAC_10_-AMPS_10_ in brine water from Uzen (bentonite 2.5 wt.%)	18.11	5.22 × 10^−4^	0.1460	0.99	Diffusion
AAm_80_-APTAC_10_-AMPS_10_ in brine water from Uzen (bentonite 5 wt.%)	7.83	0.0038	0.1300	0.98	Diffusion
AAm_90_-APTAC_5_-AMPS_5_ in brine water from Kalamkas (bentonite 2.5 wt.%)	23.23	2.04 × 10^−4^	0.0416	0.99	Diffusion
AAm_90_-APTAC_5_-AMPS_5_ in brine water from Kalamkas (bentonite 5 wt.%)	10.68	9.47 × 10^−4^	0.0622	0.99	Diffusion
AAm_80_-APTAC_10_-AMPS_10_ in brine water from Kalamkas (bentonite 2.5 wt.%)	20.11	−0.0012	0.1442	0.99	Diffusion
AAm_80_-APTAC_10_-AMPS_10_ in brine water from Kalamkas (bentonite 5 wt.%)	9.76	0.0033	0.0869	0.98	Diffusion

## Data Availability

Not available.
